# Serum Nogo - A as a predictor of neurological recovery in spinal cord injury

**DOI:** 10.6026/973206300213387

**Published:** 2025-09-30

**Authors:** Shah Waliullah, Akash Singh, Devarshi Rastogi, Deepak Kumar, Ashish Kumar, Amar Chandra Sharma, Zeenat Ara, Uday Raj

**Affiliations:** 1 Department of Orthopaedic Surgery, King George's Medical University, Lucknow, Uttar Pradesh-226003, India; 2 Department of Trauma Surgery, King George's Medical University, Lucknow, Uttar Pradesh-226003, India

**Keywords:** Spinal cord injury, neurological recovery, serum nogo-a, prognostic biomarker and therapeutic target, receptor antagonists and gene therapy

## Abstract

Spinal Cord Injury (SCI) leads to severe neurological impairment due to irreversible damage to spinal axons and limited Spinal Cord Injury (SCI) causes severe neurological impairment due to irreversible axonal damage and limited regenerative capacity. Current treatments, including surgical decompression and neuroprotective agents, have limited effectiveness, with no consensus on the optimal therapeutic combination. Anti-Nogo-A therapies show promise in preclinical studies, but their clinical efficacy remains uncertain. Emerging evidence suggests that serum Nogo-A levels may serve as prognostic biomarkers for neurological recovery in SCI patients. This study explores the potential of targeting the Nogo A/Nogo receptor pathway to enhance axonal regeneration and support the development of personalized treatment strategies.

## Background:

Spinal cord injury (SCI) leads to partial or complete loss of motor, sensory, and autonomic functions, profoundly affecting patients' quality of life and contributing to high healthcare costs [1]. Beyond paralysis and sensory deficits, SCI is often complicated by neuropathic pain, spasticity, and autonomic dysreflexia, increasing the burden on patients [2]. Predicting neurological recovery remains a major challenge, as traditional assessments like the ASIA impairment scale may not fully capture recovery complexity or predict long-term outcomes [3]. Thus, reliable biomarkers are urgently needed to guide treatment strategies. Surgical decompression within 24 hours of injury is associated with improved outcomes but is not always feasible, highlighting the need for adjunct therapies. Nogo-A, a myelin-associated protein that inhibits axonal regeneration via interaction with the Nogo receptor, has emerged as a promising biomarker and therapeutic target [4]. Combining early surgical intervention with pharmacological strategies targeting Nogo-A may enhance neural repair, offering a synergistic approach to improving functional recovery in SCI patients.

## Nogo-A: molecular structure and function:

Nogo-A, also known as reticulon 4, is a myelin-associated protein predominantly expressed in oligodendrocytes of the central nervous system (CNS). It plays a critical role in regulating axonal growth and plasticity by maintaining neural circuit stability. Structurally, Nogo-A features and N-terminal signal sequence, membrane-anchoring hydrophobic regions, and multiple loops contributing to its functional conformation. Functionally, Nogo-A inhibits axonal regeneration by binding to the Nogo receptor (NgR), activating downstream signaling pathways-primarily the RhoA/ROCK cascade-that lead to cytoskeletal reorganization and growth cone collapse. This interaction also influences calcium signaling and PKC activation, enhancing axonal growth inhibition. Following spinal cord injury (SCI), Nogo-A expression increases in glial scars and myelin debris, creating an environment that impedes axonal sprouting and reconnection [5]. By establishing inhibitory boundaries, Nogo-A limits synaptic plasticity and functional recovery was making it a key therapeutic target in SCI treatment strategies. Figure 1 (see PDF) illustrates Nogo-A's role in inhibiting axonal regeneration after spinal cord injury. Overexpressed Nogo-A binds to the NgR1 receptor complex, activate the RhoA/ROCK pathway. This signaling causes cytoskeletal changes, growth cone collapse, and axonal growth inhibition, highlighting Nogo-A as a potential biomarker and therapeutic target in SCI.

## Preclinical studies on the role of Nogo-A in spinal cord injury (SCI):

Nogo-A is a potent inhibitor of axonal regeneration and plasticity following spinal cord injury (SCI). After SCI, Nogo-A expression is markedly upregulated in the glial scar and surrounding myelin debris, creating an inhibitory environment that impedes axonal growth and functional recovery. Preclinical studies have significantly advanced our understanding of Nogo-A's role in SCI, highlighting it as a critical therapeutic target. Seminal research by [5] first identified elevated Nogo-A mRNA levels post-injury, implicating its role in axonal regeneration inhibition. Follow-up studies have demonstrated that blocking the Nogo-A/Nogo receptor (NgR) signaling pathway can promote axonal regeneration and locomotor recovery. For instance, GrandPré *et al.* [6] showed that Nogo-A receptor antagonist peptides improved outcomes in SCI rats. Similarly, Nogo-A knockout mice exhibited enhanced axonal regrowth and functional recovery compared to wild-type mice. Other preclinical studies further reinforced these findings. Sartori *et al.* [7] found improved recovery in Nogo-A-deficient mice and noted the effectiveness of NgR antagonists. Liebscher *et al.* [8] demonstrated that neutralizing Nogo-A antibodies promoted axonal sprouting and motor improvement in animal models. Moreover, Lee *et al.* [9] showed that targeting downstream effectors like the RhoA/ROCK pathway-activated by Nogo-A/NgR interaction-also enhances recovery, suggesting multiple intervention points. In addition to functional recovery, preclinical research suggests that Nogo-A may influence other injury processes. Vajda *et al.* [10] reported that Nogo-A modulates neuroinflammatory responses post-SCI, and Yasir *et al.* [11] found that it inhibits oligodendrocyte precursor cell differentiation, potentially impairing remyelination. These findings underscore the multifaceted role of Nogo-A in SCI pathophysiology. Experimental interventions targeting Nogo-A or its signaling pathways consistently enhance axonal regeneration and motor function in animal models. As a result, Nogo-A is not only a promising therapeutic target but also a potential biomarker for SCI prognosis and treatment response. Further research is essential to translate these preclinical insights into effective clinical interventions for SCI patients.

## Clinical studies on the association between Nogo-A levels and neurological outcomes in SCI:

Clinical studies have shown a significant association between elevated Nogo-A levels and poor neurological outcomes in spinal cord injury (SCI) patients. Li *et al.* [12] and Shi *et al.* [13] found that higher serum or CSF Nogo-A levels correlated with worse functional recovery, as assessed by the ASIA scale. Longitudinal observations suggest Nogo-A levels may reflect the trajectory of recovery, supporting its role as a prognostic biomarker. These findings highlight the potential of Nogo-A in guiding treatment decisions and identifying candidates for Nogo-A-targeted therapies, such as receptor antagonists or neutralizing antibodies. Further research is needed to validate Nogo-A's clinical utility in predicting and improving SCI outcomes.

## Therapeutic implications of targeting the Nogo-A/NgR pathway in spinal cord injury (SCI):

Targeting the Nogo-A/Nogo receptor (NgR) pathway offers a promising approach to promote axonal regeneration and functional recovery in spinal cord injury (SCI). Therapeutic strategies focus on disrupting the inhibitory interaction between Nogo-A and NgR through Nogo-A receptor antagonists, neutralizing antibodies, and gene therapy. Preclinical studies show that receptor antagonists and antibodies enhance axonal sprouting and improve motor function, while gene therapy targeting downstream molecules like RhoA also promotes regeneration [12, 13-14]. Despite encouraging results, translating these interventions into clinical practice faces challenges, including the complex pathology of SCI, variability in injury severity, and optimal treatment timing. Additional barriers include drug delivery to the spinal cord, potential immune reactions, and safety concerns. Strategies such as intrathecal injections and controlled dosing are being explored to improve delivery and minimize adverse effects (Table 1 - see PDF) [15]. Ongoing clinical trials are assessing safety and efficacy in humans. Continued research is essential to refine these therapies and realize their potential for improving functional outcomes in SCI patients [16].

## Limitations and future directions in understanding Nogo-A in spinal cord injury (SCI):

Despite advanced in understanding Nogo-A's role in spinal cord injury (SCI), key limitations remain. The molecular mechanisms of Nogo-A signaling and its interaction with other inhibitory factors in the injured spinal cord are not fully understood. Patient heterogeneity and a lack of standardized methods for assessing Nogo-A levels further complicate translation to clinical settings [17]. Future research should focus on clarifying downstream signaling pathways, utilizing advanced imaging to monitor recovery, and conducting well-designed clinical trials. Longitudinal studies are essential to evaluate the long-term effects of Nogo-A-targeted therapies, ultimately guiding their clinical application and improving outcomes in SCI patients.

## Future directions for clinical studies and translational research:

Future clinical studies and translational research should prioritize harnessing Nogo-A's potential as both a biomarker and therapeutic target in spinal cord injury (SCI). Biomarkers like Nogo-A may help predict neurological outcomes, guide treatment decisions, and monitor responses to therapy. Longitudinal studies are needed to evaluate its prognostic value and correlation with functional recovery [13]. Clinical trials assessing the safety and efficacy of Nogo-A-targeted therapies, including receptor antagonists and neutralizing antibodies, should involve diverse populations and standardized outcome measures. Collaborative efforts across disciplines are essential to translate preclinical findings into practice. Continued research will advance Nogo-A-based strategies and improve outcomes for SCI patients [2].

## Conclusion:

Research on Nogo-A in spinal cord injury (SCI) underscores its key role in inhibiting axonal regeneration, with elevated levels linked to poorer outcomes. As both a prognostic biomarker and therapeutic target, Nogo-A is central to emerging treatments like receptor antagonists and gene therapy. While promising, further research and multidisciplinary collaboration are needed to optimize therapies and translate findings into improved outcomes for SCI and other neurological disorders.
